# EFFECTS OF DISCLOSING DIAGNOSES AND BIOMARKER RESULTS FOR ALZHEIMER’S DISEASE AND RELATED DISORDERS

**DOI:** 10.1093/geroni/igad104.1563

**Published:** 2023-12-21

**Authors:** Takashi Amano, Brian Carpenter

## Abstract

Early diagnosis of mild cognitive impairment (MCI) and Alzheimer’s disease and related dementias (ADRD) has been recognized as a key strategy to improve health-related outcomes. However, given that these conditions are highly stigmatized, receiving a diagnostic label or knowing about biomarkers related to MCI or ADRD may have a profound impact on the person’s life. As the impacts of disclosure of diagnostic information related to MCI and ADRD on the well-being of people living with these conditions are not well understood, this symposium attempts to address this knowledge gap. The first presenter will describe a study that examined racial/ethnic variations in the effects of diagnostic labeling of ADRD on social aspects of the person’s life. The second presenter will discuss findings from a systematic review of the effects of ADRD and cognitive impairment on social engagement. The third presenter will present the findings of the study on the effect of amyloid PET results disclosure on health-related behaviors of people with MCI. The fourth presenter will describe the results of an online vignette study on the outcomes of a preclinical Alzheimer disease diagnosis. The fifth presenter will discuss the use of blood biomarkers to aid in the diagnosis of ADRD in primary care (PC). At the end of the presentation, the discussant will highlight implications for future research and policy development to alleviate negative impacts and maximize the positive impacts of a diagnostic label of MCI and ADRD.

